# A red fluorescent BODIPY probe for iridium (III) ion and its application in living cells

**DOI:** 10.1098/rsos.181090

**Published:** 2019-01-23

**Authors:** Xingyu Qu, Yongjun Bian, Jianqing Li, Yufeng Pan, Yang Bai

**Affiliations:** Department of Chemistry and Chemical Engineering, Jinzhong University, Jinzhong, Shanxi 030619, China

**Keywords:** BODIPY probe, iridium (III) ion probe, fluorescence imaging

## Abstract

A new red fluorescent probe **1** based on BODIPY skeleton has been successfully synthesized through introduction of 2-(thiophen-2-yl) quinoline moiety at *meso*- and 3-position, which exhibits excellent optical performance, including high fluorescence quantum yield, large pseudo Stokes' shift as well as high selectivity and sensitivity towards iridium (III) ion in aqueous solution and in living cells.

## 1. Introduction

Fluorescence imaging microscopy is an essential and potent tool for monitoring the analytes inside living systems based on sensitive optical response of probes to the analytes [1–5]. A fluorescence signal is characterized by its intensity, wavelength, lifetime and polarization. In fluorescence imaging, intensity used to create images can reflect the localization and concentration of the probe [6–10]. Many fluorescence probes have been synthesized to increase photo-stability and tune the emission into red or near-infrared region (NIR) (600–900 nm) to avoid photobleaching of emissive dyes and deepen penetration depths during fluorescence imaging [11–17]. BODIPY dyes show the highest potential due to many excellent features, such as the relatively high molar absorption coefficients and fluorescence quantum yields, narrow emission bandwidths with high peak intensities, the robustness against light and chemicals and accessibility of versatile dyes [18–22]. The red or NIR BODIPY probe can be easily obtained, by the introduction of aromatic or heteroaromatic rings as substituents at the 3, 5 and/or 1, 7 positions on the pyrrole moieties [23–25]. The widely used mechanisms for BODIPY probes are photoinduced electron transfer (PET), photoinduced intramolecular charge transfer (ICT) and resonance energy transfer (RET). Many BODIPY probes based on PET are often built as fluorophore–spacer–chelator constructs, in which appending the chelator at the *meso*-position of the BODIPY fluorophore decouples the two subunits [26–29]. Whereas, many BODIPY probes based on ICT often introduced the chelator in the 3, 5 and/or 1, 7 positions on the pyrrole moieties making ratiometric measurements possible [30–33].

Iridium is a rare but very useful metal that can be used in many fields including catalytic converters, luminescent materials, anti-cancer drugs, electrodes, fuel cells and jewellery etc. [34–38]*.* Especially in organic chemistry, like other heavy-metal catalysts such as palladium and platinum, iridium compounds as the catalysts could also effectively promote the transformation between various functional groups [39,40]. However, the wide use of these heavy-metals has resulted in an increase of residue levels into environment, especially ambient air and soil, which is harmful to public health. As some research findings showed, the iridium exposure could affect an immune imbalance and cause contact urticaria with anaphylactic reactions [41,42]. Thus, the oral permissible daily exposure (PDE) for iridium impurity is less than 100 µg per day by US Pharmacopeia [43]. Therefore, the recognition and detection of these heavy-metal ions have been a considerably significant and active research area. Numerous methods, particularly fluorescent methods, have been developed for the detection of palladium and platinum [44–53]. Among those, Kaur *et al.* reported the ‘off-on’ type fluorescence probe based on the BODIPY core for Pd^2+^ detection [49]. To the best of our knowledge, only an example was reported for Ir^3+^ detection to date [54]. Accordingly, exploring highly selective and sensitive probes for Ir^3+^ detection is very necessary due to the potential hazards to human health. In our previous work, we reported a BODIPY probe with a 2-(thiophen-2-yl)quinoline group as a chelator can selectively and sensitively detect the Fe^3+^ ion [55,56]. Herein, we report that functionalization of the pyrrole moieties of BODIPY with 2-(thiophen-2-yl)quinoline (TQ) tunes the dye for Ir^3+^ detection. Furthermore, fluorescence microscopy experiments demonstrated that resulting probe **1** could be used to monitor Ir^3+^ ion in living cells.

## 2. Material and methods

### 2.1. Reagents and instruments

Unless otherwise noted, the reagents or solvents were obtained from commercial suppliers and used without further purification. Metal salts, such as IrCl_3_ · 3H_2_O, RhCl_3_ · 3H_2_O, AuCl_3_, AgNO_3_, PdCl_2_, PtCl_2_, FeCl_3_ · 6H_2_O, AlCl_3_, Cd(NO_3_)_2_ · 4H_2_O, CrCl_3_, CuCl_2_ · 2H_2_O, Hg(OAc)_2_, Ni(OAc)_2_ · 4H_2_O, Pb(OAc)_2_ · 3H_2_O, Zn(OAc)_2_ · 2H_2_O, Co(OAc)_2_ · 4H_2_O, were used for making metal solutions. All air and moisture sensitive reactions were carried out under an argon atmosphere. Dry CH_2_Cl_2_ was obtained by refluxing and distilling over CaH_2_ under nitrogen. Triethylamine was obtained by simple distillation.

The ^1^H NMR and ^13^C NMR spectroscopic measurements were made by using a Bruker AVF-400 or a Bruker 700 MHz spectrometer. The measurements for ^1^H NMR and ^1^H-^1^H COSY were performed at 700 MHz (DRX-700) or 400 MHz (DRX-400). The measurements for ^13^C NMR were performed at 175 MHz (DRX-700). Mass spectra were measured with a Bruker ultraflex MALDI TOF MS spectrometer and Thermo Scientific LTQ Orbitrap XL spectrometer. Fluorescence spectral measurements were carried out by using a Hitachi F-4600 fluorescence spectrophotometer. Electronic absorption spectra were recorded with Shimadzu UV-2550 spectrophotometers.

### 2.2. Synthetic procedures for probe **1**

**Scheme 1.**
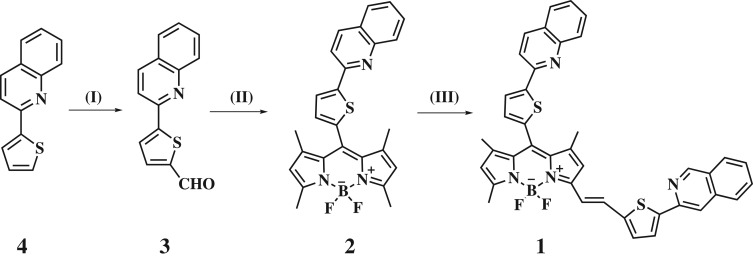
Synthetic procedures for the preparation of probe **1**. (I) *n*-butlithium and DMF in THF, –78°C (II) 2,4-dimethyl-pyrrole and trifluoroacetic acid in CH_2_Cl_2_, DDQ, triethylamine, BF_3_ · OEt_2_; (III) 5-(Quinolin-2-yl) thiophene-2-carbaldehyde, AcOH/piperidine, benzene, reflux.

The synthesis of probe **1** is outlined in scheme 1. The compounds **2** and **3** were produced according to a published procedure [55]. Probe **1** was prepared through Knoevenagel condensation of compound **2** and 5-(Quinolin-2-yl)thiophene-2-carbaldehyde **3** according to the below procedure and characterized by MALDI-TOF MS, HR-MS, ^1^H NMR, ^13^CNMR.

### 2.3. Procedures for metal ion sensing

Stock solutions of various metal ions (0.1 M), such as Ir^3+^, Rh^3+^, Au^3+^, Fe^3+^, Al^3+^, Cd^2+^, Cr^3+^, Cu^2+^, Hg^2+^, Ni^2+^, Pb^2+^, Zn^2+^, Co^2+^, Ag^+^, were prepared in deionized water. Palladium (II) solution (0.1 M) and platinum (II) solution (0.01 M) were prepared in dilute hydrochloric acid. For titration, probe **1** (3 ml, 10 µM, dimethyl sulfoxide solution) was added to the quartz optical cell of 1 cm optical path length. The metal solution was gradually added to probe **1** using a micro-pipette. For selectivity evaluation, the test samples were prepared by adding appropriate amounts of metal ion stock solution to probe **1** (3 ml, 10 µM, dimethyl sulfoxide solution). Fluorescence was measured for 590 nm excitation within 595–800 nm.

### 2.4. Cell culture and fluorescence bioimaging

The Hela cell line was provided by the Institute of Biochemistry and Cell Biology, SIBS, CAS (China). Cells were grown in high glucose Dulbecco's Modified Eagle Medium (DMEM, 4.5 g of glucose l^−1^) supplemented with 10% fetal bovine serum (FBS) at 37°C and 5% CO_2_. Cells (5 × 108 l^−1^) were plated on 14 mm glass coverslips and allowed to adhere for 24 h. Experiments to assess Ir^3+^ uptake were performed over 1 h in the same medium supplemented with 100 µM IrCl_3_ · 3H_2_O.

Immediately before the experiments, cells were washed with PBS buffer and then incubated with 5 µM probe **1** in PBS buffer for 30 min at 37°C. Cell imaging was then carried out after washing the cells with PBS buffer. Confocal fluorescence imaging was performed with a Zeiss LSM 710 laser scanning microscope and a 63× oil-immersion objective lens. Cells incubated with probe **1** were excited at 590 nm using a multi-line argon laser.

### 2.5. Theoretical calculations

Geometry optimization and TD-DFT calculations were carried out for probe **1** and probe **1** with Ir^3+^ using the B3LYP functional of the Spartan 16 software packages with 6-31G(d) basis sets.

## 3. Results and discussion

### 3.1. Characterization of the probe **1**

**Figure 1.**
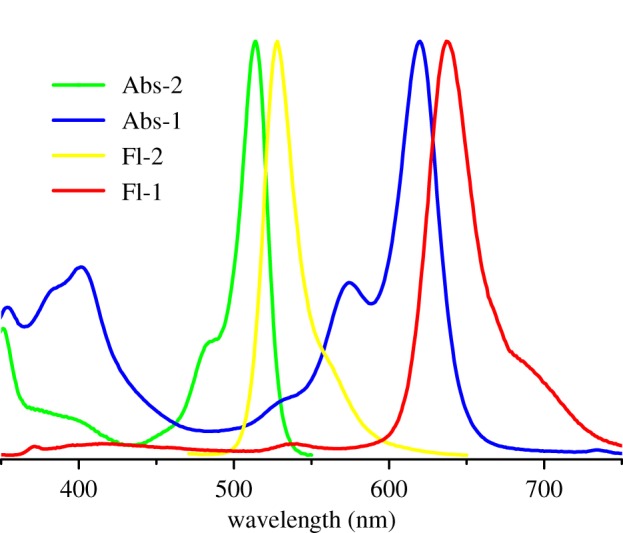
UV–Vis absorption and fluorescence spectra of probe **1** (10 µM) (red line) and compound **2** (10 µM) (green line) in dichloromethane.

Figure 1 shows the UV/visible absorption and emission spectra of compounds **1** and **2** in CH_2_Cl_2_. The absorption maximum of compound **2** is centred at approximately 514 nm. By contrast, the absorption maxima of probe **1** is red shifted to 625 nm mainly due to the introduction of a TQ substituent at 3-position on the pyrrole moiety. Exciting the 2-(thiophen-2-yl)quinoline moiety at 334 nm in compound **4** provides an emission band at 386 nm. Whereas the emission from the TQ moiety in probe **1** is almost quenched completely upon excitation at 334 nm, strong emissions typical for the BODIPY fluorescence at 635 nm for probe **1** are observed. The fluorescence quantum yield of probe **1** in dimethyl sulfoxide (DMSO) is 0.34 and the pseudo Stokes' shift is up to 300 nm. These results indicate that probe **1** has a large pseudo Stokes' shift and efficient through-bond energy transfer from the TQ donor to the BODIPY acceptor.

### 3.2. Fluorescence detection and selectivity of probe **1**

**Figure 2.**
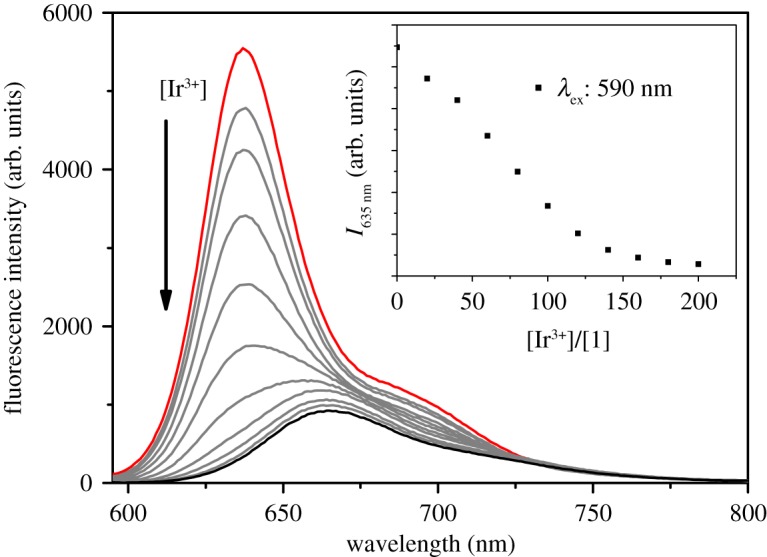
The fluorescence spectrum of probe **1** is (10 µM in DMSO) in the presence of different Ir^3+^ concentration. (Inset) The emission intensity at 635 nm of probe **1** changes as a function of the Ir^3+^ concentration.

The response of probe **1** with Ir^3+^ is investigated by spectrophotometric titration in DMSO, as shown in figure 2. After adding Ir^3+^ from 0 to 100 eq ([metal]/[probe **1**]), the fluorescence intensity of probe **1** at 635 nm decreased remarkably with a virtually unchanged peak position. Upon addition of more than 100 equiv. of Ir^3+^, the red shift in the fluorescence peak of probe **1** about 30 nm can be observed. As shown in electronic supplementary material, figure S9, the fluorescence response of probe **1** towards Ir^3+^ is normalized as *R* = 1 − *I*_i_/*I*_max_, where *I*_max_ is fluorescence response of probe **1** without Ir^3+^, *I*_i_ is luminescent intensity of probe **1** with Ir^3+^ [47]. The detection limit of probe **1** for Ir^3+^ is determined as 2.55 ppm based on the signal-to-noise ratio of three. The good linear relationship between the fluorescence response and concentration of Ir^3+^ is obtained with a 0.9910 correlation coefficient.

**Figure 3.**
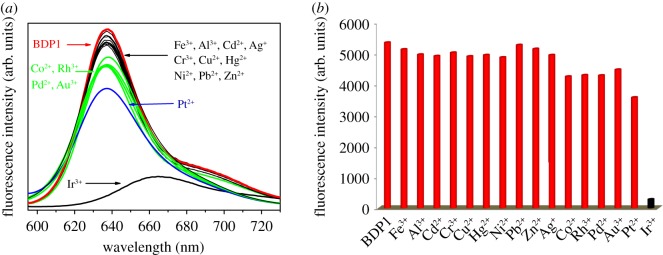
(*a*) Fluorescence spectra of 10 µM probe **1** upon addition of different metal ions in DMSO solution. (*b*) The fluorescence intensity of probe **1** is observed at 635 nm upon addition of various anions.

To gauge the recognition specificity of probe **1** to Ir^3+^ ion, the spectral response features of probe **1** to various interference metal ions with much higher concentration were estimated. The selectivity of probe **1** with Ir^3+^ is examined according to its bonds with various metal ions as shown in figure 3. When the metal ions ([metal]/[probe **1**] = 200:1), such as Fe^3+^, Al^3+^, Cd^2+^, Ag^+^, Cr^3+^, Cu^2+^, Hg^2+^, Ni^2+^, Pb^2+^, Zn^2+^, are added to the probe **1** solution (10 µM in DMSO), the fluorescence spectrum peak and intensity is nearly unchanged. After the addition of the metal ions (Co^2+^, Rh^3+^, Pd^2+^, Au^3+^, Pt^2+^, Ir^3+^) under the same conditions, the fluorescence intensity decreased by 20%, 20%, 20%, 16%, 33%, 94%, respectively. The above results demonstrate that its Ir^3+^ response is not interfered in the background containing appropriate metal ions.

### 3.3. Sensing mechanism of probe **1** with Ir^3+^

**Figure 4.**
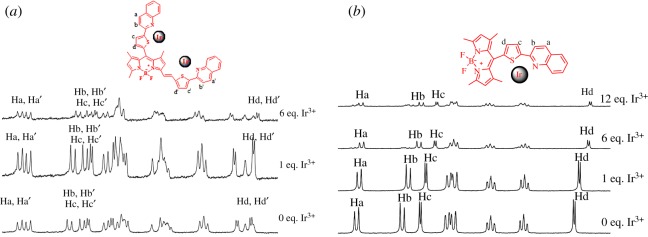
^1^H NMR of probe **1** (*a*) and compound **2** (*b*) with different concentrations of Ir^3+^ in DMSO-d_6_.

That probe **1** could selectively monitor Ir^3+^ ion was possible because of the stable binding with sulfur and nitrogen atom in TQ parts. To further demonstrate this mechanism, the binding mode of Ir^3+^ ion with probe **1** or compound **2** is investigated by ^1^H NMR spectroscopy as shown in figure 4 and electronic supplementary material, figure S7. The ^1^H NMR spectra of probe **1** and compound **2** in the presence of different concentrations of Ir^3+^ are recorded in DMSO-d_6_ and compared to the spectrum of the ion-free probe. During ^1^H NMR spectra of compound **2**, the protons (Hc and Hd) of the thiophene group at 8.14 ppm and 7.32 ppm are affected significantly by Ir^3+^. Upon the addition of Ir^3+^, significant downfield shifts of protons (*Δ*_Hc_ = 0.08 ppm) and (*Δ*_Hd_ = 0.07 ppm) corresponding to the protons of the thiophene group are observed, indicating that Ir^3+^ coordinates to the sulfur atom of compound **2**. Furthermore, the proton (Hb) of the quinoline group at 8.24 ppm exhibits a downfield shift (*Δ*_Hb_ = 0.09 ppm), indicating that Ir^3+^ coordinates to the nitrogen atom of compound **2**. The results indicate that the sulfur atom and nitrogen atom of compound **2** may be involved in coordination to Ir^3+^. Figure 4 shows that the protons (Hc, Hb, Hd and Hc′, Hb′, Hd′) of probe **1** have significant downfield shifts as well, which indicate that probe **1** and compound **2** have similarities to binding of Ir^3+^ ion.

Probe **1** based on oxidative PET is in the fluorescence ‘on’ state when in the ion-free form. Coordination of TQ moiety at *meso*-position of probe **1** with Ir^3+^ ion makes it electron poor and lowers the LUMO of TQ moiety. Our theoretical calculation revealed that the LUMO orbital energy of probe **1** with Ir^3+^ is lower, compared to the probe **1**(−2.68 eV versus −4.01 eV) (electronic supplementary material, figure S8), so that PET from the excited BODIPY moiety to the TQ moiety at *meso*-position becomes possible, leading to fluorescence quenching. Probe **1** possesses an electron-donating group (TQ moiety at 3-position) conjugated to an electron-withdrawing group (BODIPY moiety); it undergoes ICT from the donor to the acceptor upon photo-excitation. The interaction of Ir^3+^ ion with the TQ moiety at 3-position enhances the electron-withdrawing character and consequently red shifted the fluorescence spectra.

### 3.4. Practical application of probe **1**

**Figure 5.**
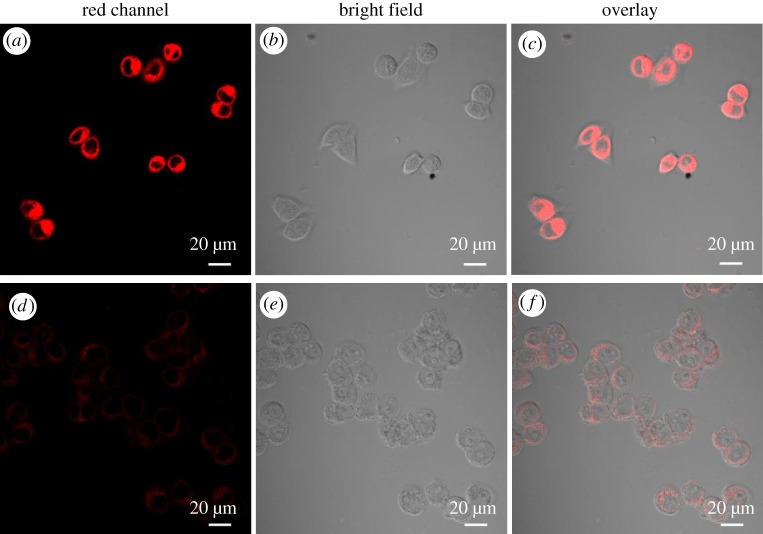
Confocal fluorescence, bright field images and overlay image of probe **1** in Hela cells. (*a*) Cells incubated with 5 µM of probe **1** for 30 min at 37°C upon excitation at 590 nm. (*d*) Cells supplemented with 100 µM of Ir^3+^ in the growth media for 1 h at 37°C upon excitation at 590 nm. (*b*,*e*) Bright field image of cells shown in panel. (*d*,*f*) Overlay image of cells shown in panel.

The practical application of probe **1** as Ir^3+^ probe in living cells has been evaluated. As shown in figure 5, after incubating the Hela cells with 5 µM probe **1** for 30 min at 37°C, a significant green emission from the intracellular region could be observed upon excitation at 590 nm (figure 5*a*). Supplementing cells with 100 µM Ir^3+^ in the growth medium for 1 h at 37°C, microscope images showed only weak intracellular fluorescence (figure 5*d*). Both bright field and overlay measurements with Ir^3+^ and probe **1** after treatment confirmed that the cells are viable throughout the imaging experiments (figure 5*b*,*c*,*e*,*f*). In addition, large signal ratios (I2/I1 > 20) are observed at the cytoplasm (region 2) and the nucleus (region 1) due to weak nuclear uptake of probe **1** (electronic supplementary material, figure S10). These data lead to exclusive staining in the cytoplasm, which indicates that probe **1** is cell-permeable. These results indicate that probe **1** can be used as an effective intracellular Ir^3+^ imaging agent.

**Figure 6.**
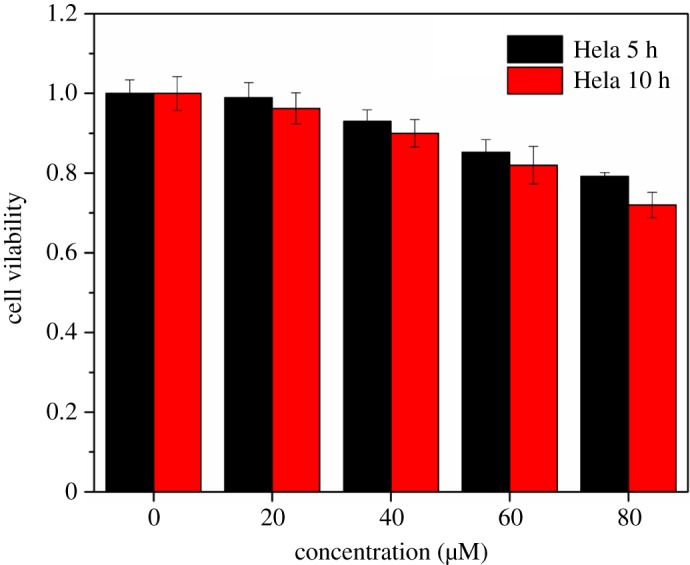
MTT assay of probe **1**.

The cell viability of probe **1** is evaluated through an MTT assay (figure 6). Cell viability is monitored for 5 and 10 h after treatment with probe **1** over a wide range of concentrations (0–80 µM). This demonstrates that probe **1** does not negatively affect the cell viability over the range of concentrations studied, therefore, can clearly be used for the intracellular detection of the Ir^3+^ ion.

## 4. Conclusion

In summary, we have designed a red BODIPY probe with 2-(thiophen-2-yl)quinoline substituent at *meso*- and 3-position, which exhibits excellent optical performance with a pseudo Stokes' shift (300 nm) and strong emission. The high selectivity and sensitivity of probe **1** towards Ir^3+^ indicate that probe **1** can be used as a fluorescence turn-off sensor for Ir^3+^ in living cells. Probe **1** in fluorescence imaging can avoid photobleaching of emissive dyes and deepen penetration depths. This study displays an effective rationale to design chemosensors for rapid Ir^3+^ sensing in biological systems. Meanwhile, there is still considerable scope for further research in this area for the detection of biologically noble metals.

## 5. Experimental

### 5.1. General procedure for synthesis of compound **2**

Compound **2** is synthesized according to the published procedures [55]. Red solid; 65% yield. ^1^H NMR(DMSO-d_6_, 400 MHz): 8.48 (d, 1H, *J* = 8 Hz), 8.24 (d, 1H, *J* = 8 Hz), 8.14 (d, 1H, *J* = 4 Hz), 8.00 (q, 2H, *J* = 8 Hz), 7.77 (t, 1H, *J* = 12 Hz), 7.59 (t, 1H, *J* = 12 Hz), 7.33 (d, 1H, *J* = 4 Hz), 6.26 (s, 2H), 2.47 (s, 6H), 1.72 (s, 6H).

### 5.2. General procedure for synthesis of probe **1**

Compound **2** (0.22 mmol, 100 mg) and compound **3** (0.26 mmol, 63 mg) are added to a 50 ml round-bottomed flask containing 20 ml benzene solution under an argon atmosphere. And then piperidine (0.4 ml) and acetic acid (0.4 ml) are added to this solution using a syringe. The mixture is heated under reflux by using a Dean Stark trap and reaction was monitored by TLC method. After the starting compound **2** disappearing, the mixture is cooled to room temperature. Then the reaction mixture is washed with water, dried over Na_2_SO_4_, and concentrated at reduced pressure. The crude product is purified by silica-gel column chromatography using CH_2_Cl_2_ as the eluent to afford the black power **1**. Yield: 5.5%. ^1^H NMR(CDCl_3_, 700 MHz): 8.20 (d, 1H, *J* = 7 Hz), 8.16 (d, 1H, *J* = 14 Hz), 8.12 (d, 1H, *J* = 7 Hz), 8.09 (d, 1H, *J* = 7 Hz), 7.85 (d, 1H, *J* = 7 Hz), 7.81–7.78 (m, 5H), 7.72 (t, 1H, *J* = 7 Hz), 7.68 (s, 1H), 7.62 (d, 1H, *J* = 21 Hz), 7.51 (m, 2H), 7.39 (d, 1H, *J* = 14 Hz), 7.07 (s, 1H), 6.65 (s, 1H), 6.07 (s, 1H), 2.64 (s, 3H), 1.81 (s, 3H), 1.78 (s, 3H). ^13^C NMR(CDCl_3_, 175 MHz): 156.61, 152.23, 151.76, 151.59, 146.21, 148.04, 147.63, 146.49, 144.92, 143.34, 142.47, 137.80, 136.88, 136.60, 133.88, 132.93, 131.85, 130.14, 130.03, 129.42, 129.34, 129.29, 129.15, 129.09, 127.56, 127.52, 127.38, 127.32, 126.75, 126.53, 126.34, 125.81, 121.88, 119.33, 118.12, 117.59, 117.23, 29.75, 14.24, 14.13. MALDI-TOF MS, calcd. for C_40_H_29_BF_2_N_4_S_2_: 678.18948; found: 678.208 [M^+^]. HR-MS, calcd. for C_40_H_29_BF_2_N_4_S_2_: 678.18948; found: 678.18976 [M^+^].

## Supplementary Material

Supplementary Figures

## Data accessibility

The spectroscopic data are submitted to journal as supporting files. The supplementary data are available at http://dx.doi.org/10.6084/m9.figshare.6795095.
