# Supplementary material for: A red fluorescent BODIPY probe for iridium (III) ion and its application in living cells
Source: R Soc Open Sci. 2019 Jan 23;6(1):181090. doi: 10.1098/rsos.181090 (PMC6366194; doi:10.1098/rsos.181090)
Supplement: Supplementary Figures [file rsos181090supp1.docx]

***Supporting Information***

**A red fluorescent BODIPY probe for iridium (III) ion and its application in living cells**

*Xingyu Qu, Yongjun Bian,^*^ Jianqing Li, Yufeng Pan and Yang Bai*

*Department of Chemistry and Chemical Engineering, Jinzhong University, Jinzhong, Shanxi, 030600, China*

*corresponding authors E-mail addresses: yjbian2013@jzxy.edu.cn*

**Table of Contents**

Supplementary data

Figure S1. ^1^H NMR spectrum of compound **2**.

Figure S2. ^1^H-^1^H COSY spectrum of compound **2**.

Figure S3. ^1^H NMR spectrum of probe **1**.

Figure S4. ^13^C NMR spectrum of probe **1**.

Figure S5. HR-MS spectrum of probe **1**.

Figure S6. MALDI-TOF MS spectrum of probe **1**.

Figure S7. The full ^1^H NMR spectrum of probe **1** (left) and compound **2** (right) with different

concentrations of Ir^3+^ in DMSO-d_6_.

Figure S8. Frontier orbital energy of probe **1** and probe**1** with Ir^3+^

Figure S9. Dependence of response signal on concentration of Ir^3+^ ions

Figure S10. Confocal fluorescence imaging of living HeLa cells incubated with probe **1**


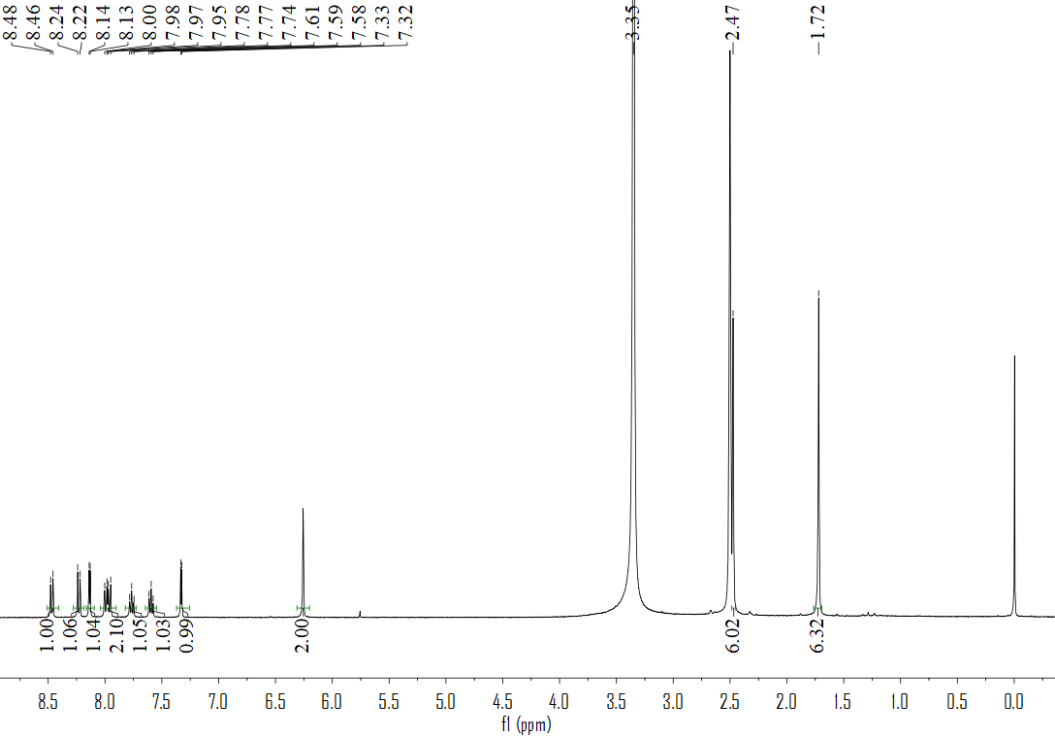


**Fig. S1** ^1^H NMR spectrum of compound **2** in DMSO-d_6_


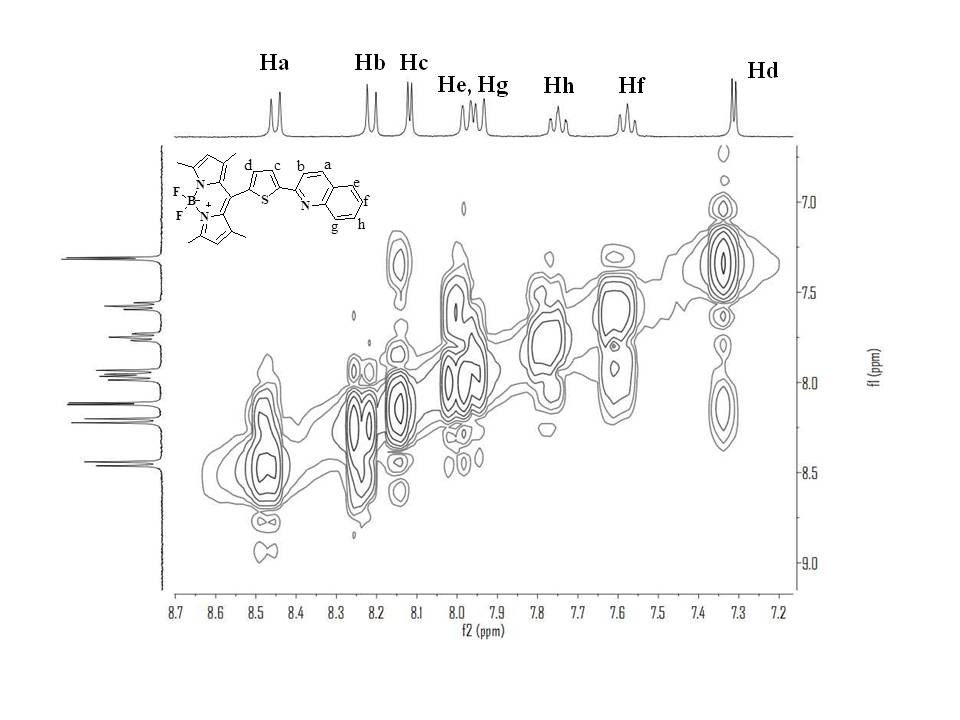


**Fig. S2** ^1^H-^1^H COSY spectrum of compound **2** in DMSO-d_6_


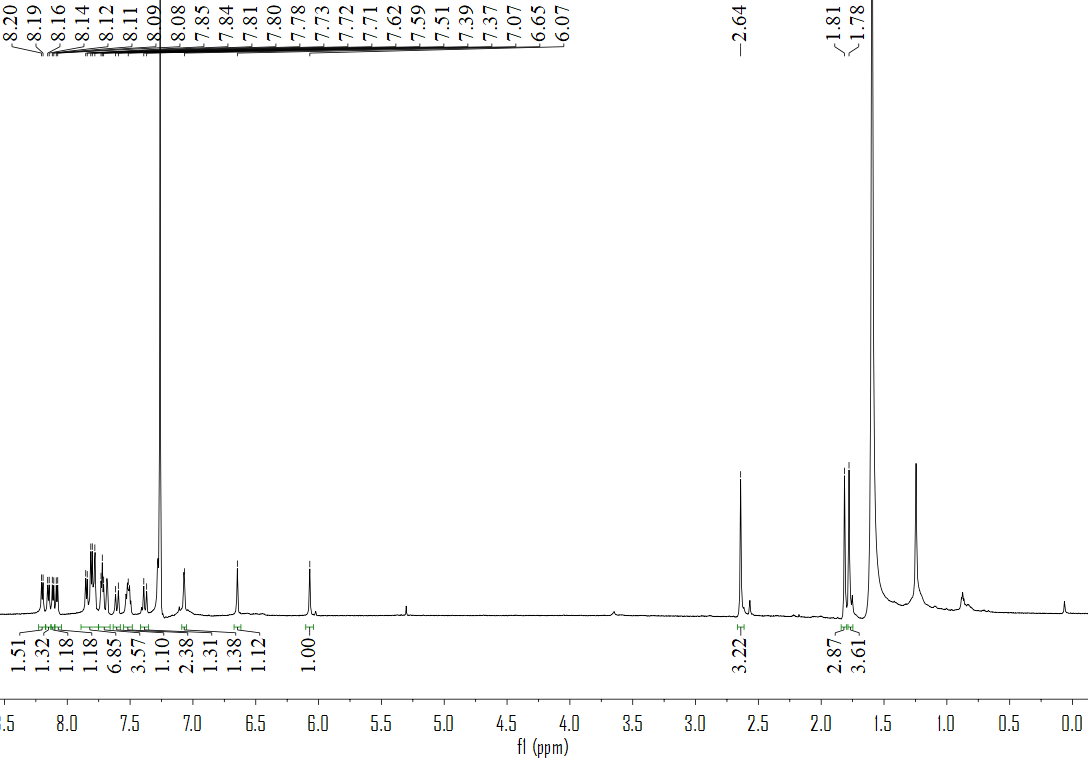


**Fig. S3** ^1^H NMR spectrum of probe **1** in CDCl_3_.


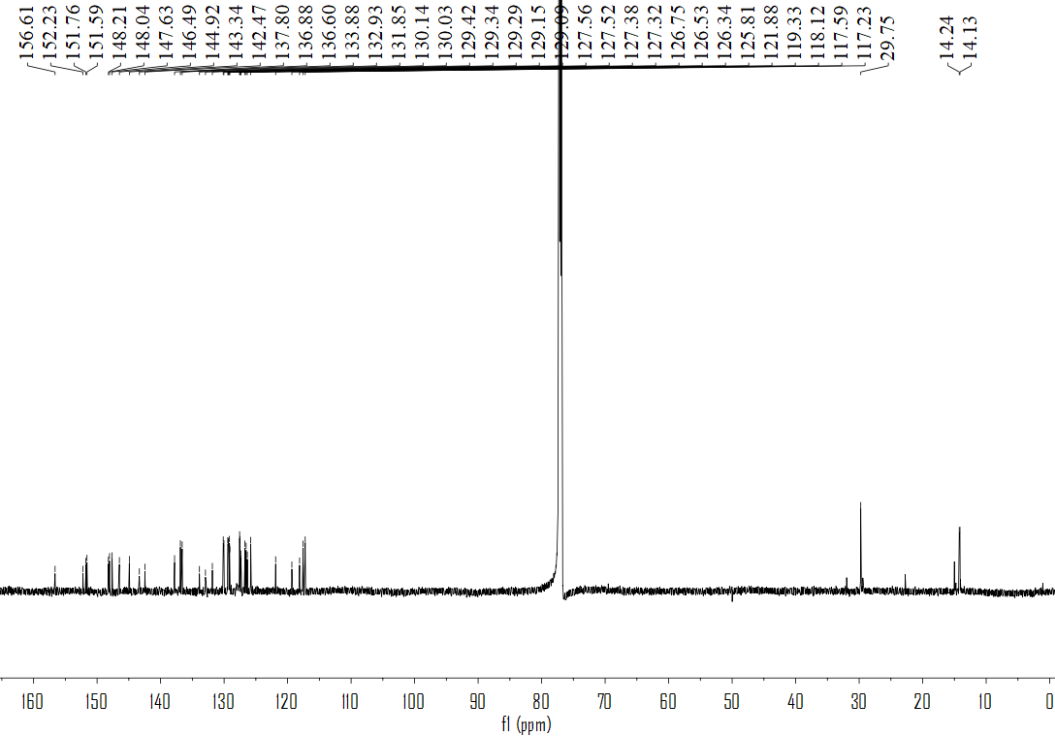


**Fig. S4** ^13^C NMR spectrum of probe **1** in CDCl_3_.

**Fig. S5** HR-MS spectrum of probe **1**.

**Fig. S6** MALDI-TOF MS of probe **1**.

**
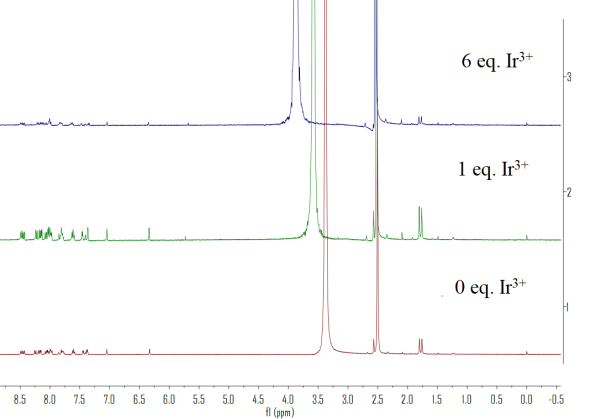

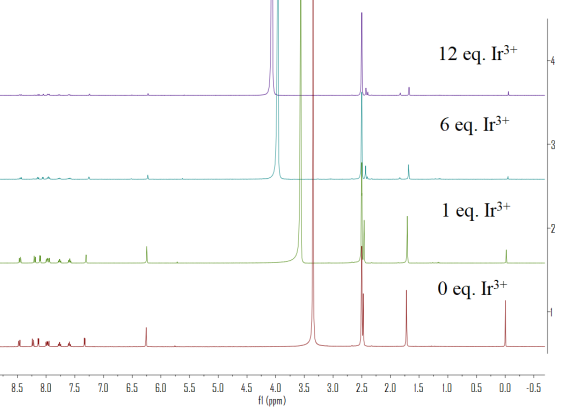
**

**Fig. S7** The full ^1^H NMR spectrum of probe **1** (left) and compound **2** (right) with different concentrations of Ir^3+^ in DMSO-d_6_.

**
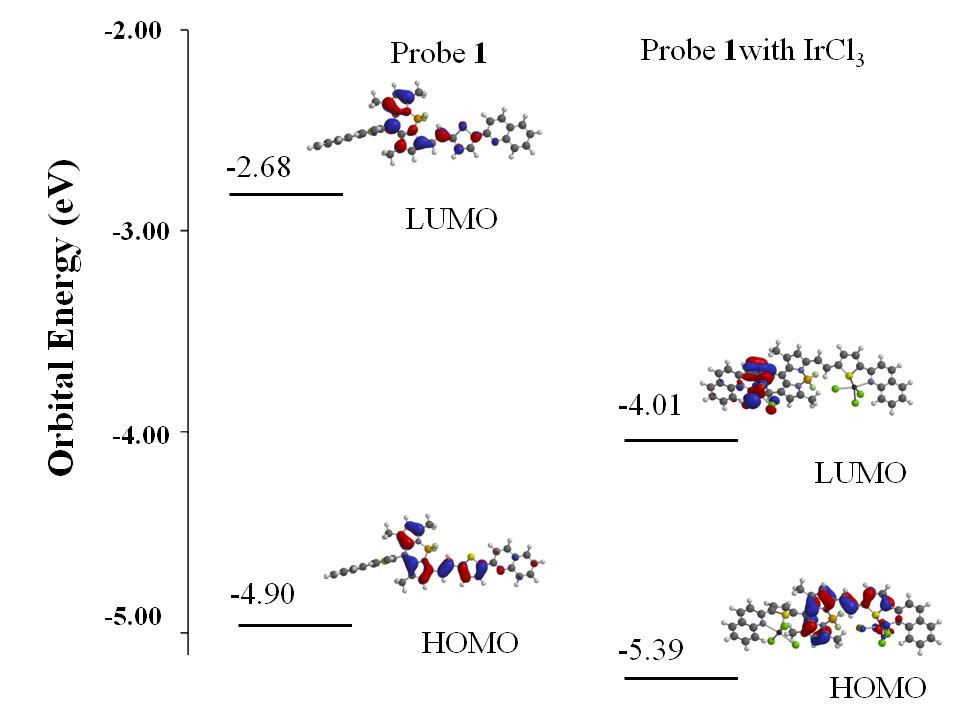
**

**Fig. S8** Frontier orbital energy of probe **1** and probe **1** with Ir^3+^ calculated with B3LYP level 6-31G(d) basis sets

**
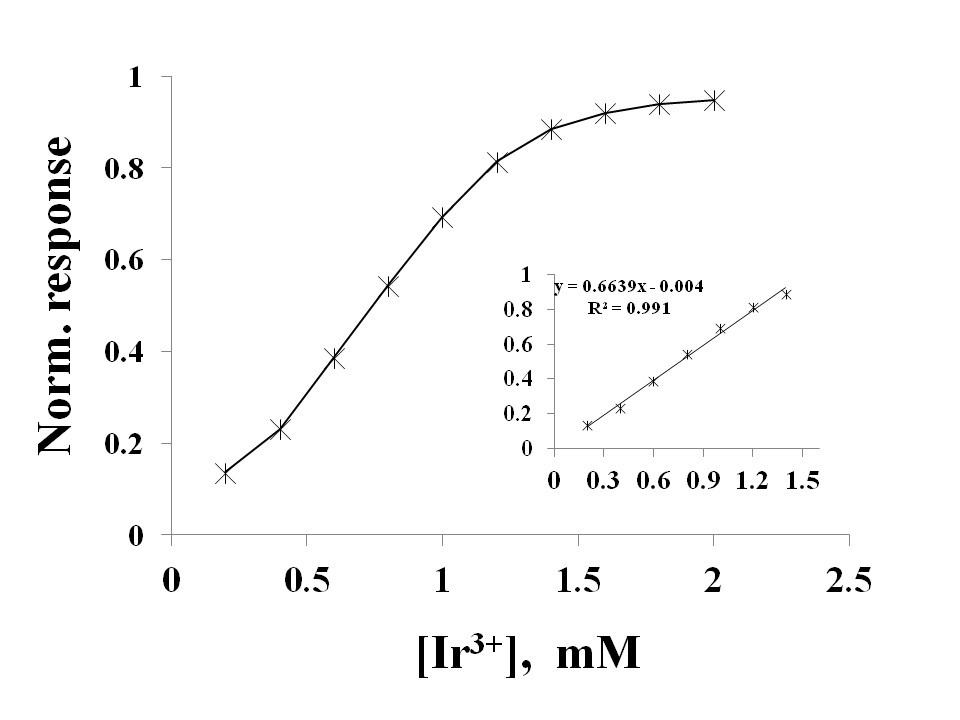
**

**Fig. S9** Dependence of response signal on concentration of Ir^3+^ ions (λex = 590 nm, λem = 635 nm).

**
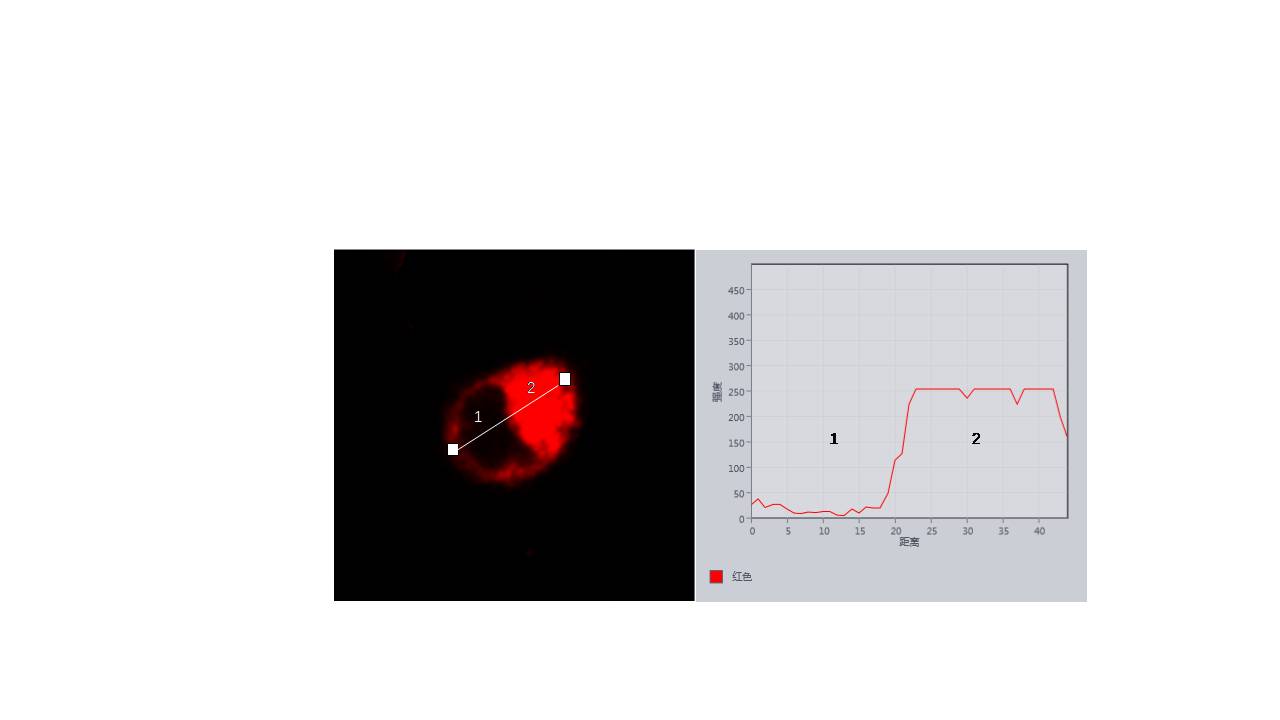
**

**Fig. S10** (A) Confocal fluorescence imaging of living HeLa cells incubated with 5μM probe **1** for 30 min at 25 ºC. (B) Normalized fluorescence intensity proﬁle across the line shown in panel A corresponding to nuclear region (1) and cytoplasm (2)
